# Plasma miRNAs as potential biomarkers of chronic degenerative valvular disease in Dachshunds

**DOI:** 10.1186/s12917-014-0205-8

**Published:** 2014-09-26

**Authors:** Magdalena Hulanicka, Magdalena Garncarz, Marta Parzeniecka-Jaworska, Michał Jank

**Affiliations:** Department of Physiological Sciences, Faculty of Veterinary Medicine, Warsaw University of Life Sciences, Nowoursynowska str. 159c, 02-776 Warsaw, Poland; Department of Veterinary Diagnostics and Pathology, Faculty of Veterinary Medicine, Warsaw University of Life Sciences, Nowoursynowska str. 159c, 02-776 Warsaw, Poland

**Keywords:** Dachshunds, Endocardiosis, miRNA, Plasma, ACVIM, Dog, qPCR

## Abstract

**Background:**

Endocardiosis is the most common heart disease in Dachshunds and is therefore an important cause of cardiac morbidity and death. In recent years we have observed an increasing interest in the development of new genetic and genomic markers of heart disease. The discovery of miRNAs circulating in biofluids such as plasma or serum aroused researchers’ interest in using them as potential biomarkers. In the present study we analysed the expression of 9 miRNAs described in literature as being involved in cardiovascular pathology in the plasma of dogs suffering from endocardiosis.

**Results:**

Expression analysis using the Real-time PCR method revealed that two out of nine miRNAs were significantly downregulated: the expression of miR-30b differed between ACVIM stage B and stage A (control) dogs; the expression of mi-133b differed ACVIM stage C and stage A dogs. 5 miRNAs (miR-125, miR-126, miR-21, miR-29b and miR-30b) showed a trend of downregulation in the ACVIM C group. Levels of miR-423 were the same in healthy and diseased dogs. Expression of miR-208a and 208b was not detected.

**Conclusions:**

miR-30b could be a potential biomarker of ACVIM stage B heart failure in Dachshunds with endocardiosis and miR-133b could be a potential biomarker of ACVIM stage C. The lack of expression or lack of significant changes in expression in 7 miRNAs which are potential biomarkers of heart diseases in humans proves that findings from human medicine are not always directly reflected in veterinary medicine.

**Electronic supplementary material:**

The online version of this article (doi:10.1186/s12917-014-0205-8) contains supplementary material, which is available to authorized users.

## Background

Endocardiosis is the most common heart disease in Dachshunds and is therefore an important cause of cardiac morbidity and death. It is recognised mainly in middle-aged and older dogs. It is also referred to as myxomatous atrioventricular valvular degeneration, chronic degenerative valvular heart disease (CVHD), or chronic valvular fibrosis. Endocardiosis is characterised by progressive lesions affecting primarily the mitral valve, and less frequently the tricuspid valve. Affected valve leaflets are grossly shortened and thickened and have nodular areas along the free valvular edges. During disease progress, lesions extend and occupy larger areas of the valve surface, sometimes encompassing the chordae tendineae. In most cases atrioventricular valvulopathy occurs in the dog spontaneously and is age-related [1-3].

In recent years significant development of new diagnostic methods allowing precise diagnosis of animal diseases as well as better understanding of their background has been observed. Genetics is a very important aspect of dog breeding and reproduction, and another significant reason why we have been observing a great interest in the development of new genetic and genomic markers of specific diseases. One group of such markers could be miRNA.

miRNAs inhibit gene expression at the post-transcriptional level by directing the RISC complex to target mRNAs resulting in translational repression or cleavage [4]. The transcription profile of many genes can be influenced by miRNAs. This is why they have emerged as crucial regulators of a wide range of physiological and pathological processes.

Several studies have recently discovered circulating miRNAs in blood, not only in platelets, nucleated blood cells, and erythrocytes, but also in plasma. Moreover, plasma miRNAs were found to be unexpectedly stable even under conditions such as boiling, long time storage at room temperature and low or high pH, whereas exogenously added synthetic miRNAs are quickly degraded because of RNAse activity in the plasma [5-7]. This means that endogenous miRNAs present in plasma must be shielded in some way from degradation. Recent studies have showed that miRNAs are packaged in lipid vesicles (exosomes, microvesicles, apoptotic bodies) or associated with RNA-binding proteins or lipoprotein complexes [8-12]. These properties make miRNAs ideal stable novel diagnostics biomarkers, which can be measured in easy accessible samples and can be used to diagnose different pathological conditions. Plasma and serum miRNAs are currently being intensively investigated and specific miRNA expression patterns are being reported for various pathological conditions in humans. The results of recent studies show that circulating miRNAs are potential biomarkers for detection of cancer [13-16] and heart disease [17-19].

However, in veterinary medicine the situation is quite different–there are only few reports about research on circulating miRNAs research in animal diseases. To our best knowledge there is only one report concerning circulating miRNA levels in dogs with heart disease. This study compares expression of miRNAs in the serum of Doberman Pinschers with dilated cardiomyopathy (DCM) in comparison to healthy control dogs.

Therefore the aim of the present paper was to compare levels of miRNAs in the plasma of Dachshunds with endocardiosis and healthy control dogs. Moreover, dogs with endocardiosis were divided in two groups according to the ACVIM (American College of Veterinary Internal Medicine) classification scheme [20]. The results of our previous study revealed that the biggest differences in expression of genes in peripheral blood nuclear cells were observed when healthy dogs and ACVIM stage C (mild to moderate heart failure) dogs where compared, so we decided to include the ACVIM stage C group in the present study. Furthermore, we also included the ACVIM stage B group in our study, which is a group of great importance since this is the asymptomatic stage of the disease and deemed to be important for early diagnostics. The potential identification of any prognostic marker of canine endocardiosis would be very important for the staging of disease progress, therapy assessment as well as for breeding purposes. It could give new insight into the pathogenesis and staging of the disease which could help in the effective planning of treatment.

## Results

### Patient characteristics

24 client-owned Dachshunds were examined cardiologically in the Cardiology Service of the Small Animal Clinic, Faculty of Veterinary Medicine, School of Agriculture in Warsaw. Dogs included in the study were assigned to ACVIM B (n = 8), ACVIM C (n = 8) or ACVIM A–unaffected control group (n = 8) according to the ACVIM (American College of Veterinary Internal Medicine) classification scheme. One sample of the ACVIM C group was removed from further analysis because of inconsistency of expression levels of miRNA compared to other samples. Study population characteristics are shown in Table 1.

**Table 1:** **Clinical characteristics of the study population in the control, ACVIM B and ACVIM C groups**

**ACVIM stage**		**ACVIM A–unaffected (mean ± SD)**	**ACVIM B (mean ± SD)**	**ACVIM C (mean ± SD)**
Numer of dogs		8	8	7
Age range (years)		5.458 ± 2.51	10.17 ± 3.36	10.40 ± 4.73
Sex	Males	2	4	5
	Females	6	4	3
Body weight (kg)		10.38 ± 3.26	9.46 ± 2.13	9.20 ± 1.83
EF %		71.25 ± 12.26	69.5 ± 8.21	77.14 ± 3.39
LA/Ao		1.223 ± 0.10	1.479 ± 0.41	1.774 ± 0.21*
LA cm		1.938 ± 0.47	2.348 ± 0.54	2.811 ± 0.32*
LVDd		2.863 ± 0.24	2.993 ± 0.43	3.529 ± 0.42**
MVR		0	5.466 ± 1.05*** (n = 5)	5.963 ± 0.78***
RVDd		0.9375 ± 0.17	0.9275 ± 0.14	0.6057 ± 0.21**
TVR m/s		0.3988 ± 1.13	0.925 ± 1.39	2.379 ± 1.19*

*statistically significant difference vs healthy dogs with p < 0.05; **statistically significant difference vs healthy dogs with p < 0.01; ***statistically significant difference vs healthy dogs with p < 0.001.

### qPCR

**Figure 1:**
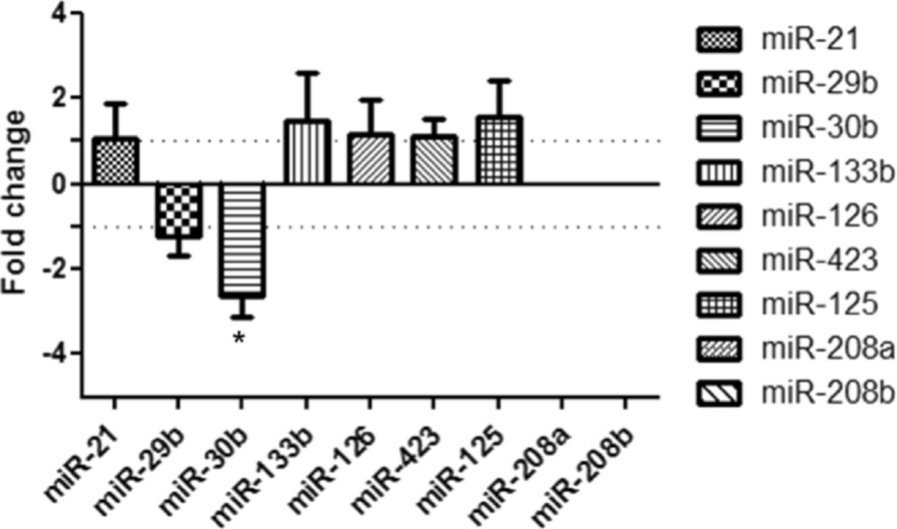
**The expression of 9 miRNAs in dogs with ACVIM stage B of heart failure in comparison to unaffected control (fold changes relative to healthy group; mean ± SEM).**

Nine miRNAs which have been previously described in literature as being involved in cardiovascular pathology in humans were selected for qPCR analysis (Table 2). The expression of each miRNA in the plasma of dogs was compared between the ACVIM stage B group and unaffected dogs (ACVIM stage A) using parametric t-test and Bonferroni correction. The results revealed that miR-30b was significantly downregulated in ACVIM stage B–its expression was -2.63 times lower than in unaffected dogs. The rest of analysed miRNAs showed almost no differences between these two groups. Moreover, results showed a high interindividual variance of expression. miR-208a and miR-208b were not detected in any of the examined samples (in both healthy group of dogs–ACVIM stage A and ACVIM stage B) (Figure 1).

**Table 2:** **Overview of miRNAs in various cardiovascular diseases in humans, rats and mouse**

**microRNA**	**Disease**	**Material**	**Regulation**	**Species**	**Author**
miR-30	Chronic Systolic Heart Failure	Serum	↑	Human	[21]
miR-21	Dilated Cardiomyopathy	Heart tissue	↑	Human	[22]
miR-29	Acute Myocardial Infarction	Heart tissue	↓	Human and mice	[23]
miR-126	Congestive Heart Failure	Plasma	↓	Human	[18]
	Coronary Artery Disease	Plasma	↓	Human	[24]
	Dilated Cardiomyopathy	Heart tissue	↓	Human	[22]
miR-125	Ischemic Dilated Cardiomyopathy	Peripheral blood mononuclear cells	↓	Human	[25]
miR-423	Heart Failure	Plasma	↑	Human	[26]
	Heart Failure	Serum	↑	Human	[21]
miR-208	Acute Myocardial Infarction	Plasma	↑	Human	[17]
	Myocardial Injury	Plasma	↑	Rat	[27]
miR-133	Myocardial Infarction	Heart tissue	↓	Human	[28]

**Figure 2:**
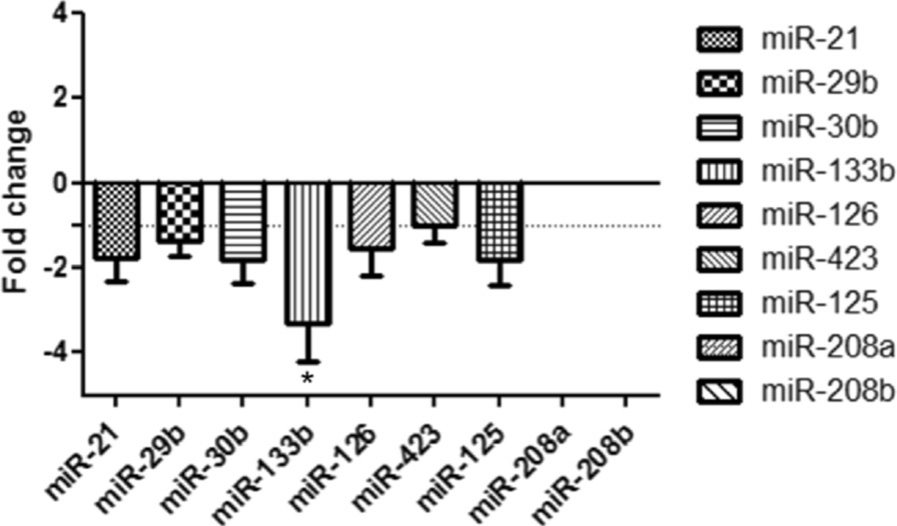
**The expression of 9 miRNAs in dogs with ACVIM stage C of heart failure in comparison to unaffected control (fold changes relative to normal group; mean ± SEM).**

Using the same statistical tests the expression of miRNAs in ACVIM stage C and unaffected dogs was analysed. We observed a trend of downregulation in all examined miRNAs. The biggest differences were observed in case of miR-133b. The expression of this miRNA was -3.29 times lower in ACVIM stage C than in unaffected dogs. miR-29b and miR-423 remained almost unchanged but the other miRNAs were slightly downregulated. The mean fold change for each of them was as follows: -1.84 for miR-30b, -1.55 for miR-126, -1.82 for miR-125. Likewise in ACVIM stage B class the expression of miR-208a and miR-208b were not detected (Figure 2).

## Discussion

This study reports the results of an evaluation of the expression of miRNAs as potential biomarkers in the plasma of dogs suffering from endocardiosis. Endocardiosis (also referred to as chronic degenerative valvular disease or myxomatous valve disease) can lead to congestive heart failure and the associated clinical signs. Since the molecular background of this disease is still far from fully elucidated, the search for any specific markers (prognostic, therapeutic) of this disease would be of great value and importance.

Although miRNA are currently intensively investigated in human medicine because of their diagnostic potential in many different conditions, there are only few reports related to circulating miRNA studies in animal diseases. One of these is the study about expression of miRNAs in serum of Doberman Pinschers with dilated cardiomyopathy (DCM). The authors compared the expression of 1368 miRNAs using miRNA microarrays. They identified 404 different miRNAs but the results did not show statistical significance after multiple testing correction (FDR) and Real-time PCR validation of 5 miRNAs which were differentially regulated without FDR correction showed no statistical differences [29]. Another study conducted by Mizuno et al. revealed that the level of muscle-specific miRNAs are inceresed in serum of canine X-linked muscular dystrophy in Japan dog model (CXMDJ) in comparison to wild type control dogs [30]. Moreover, there were miRNAs quantitated in peripheral blood from dogs with chronic lymphocytic leukemia. The results revealed distinct miRNA patterns in chronic lymphocytic leukemia depending on the immunophenotype [31]. The number of studies is not sufficient and much more data are needed to determine whether miRNAs can be used as biomarkers in veterinary medicine.

One of the difficulties researchers face related to circulating miRNA studies is the impossibility of reliable large–scale expression screening using the microarray method. Microarray analysis is not a suitable method for studying miRNAs expression in biofluids because of insufficient sensitivity which cannot ensure reproducible detection of samples with low miRNA levels [18]. The results of a study conducted by Jensen et al. revealed that the best specificity, sensitivity reproducibility, recovery and linearity in circulating miRNA studies is attained with the Real-time PCR method, which is based on Locked Nucleic Acid (LNA™) technology [32]. This is why we decided to apply this method in our analysis.

Our study included 24 client-owned dogs with endocardiosis classified into groups according to the ACVIM (American College of Veterinary Internal Medicine) classification scheme. The results revealed that among nine miRNAs that have been previously connected to heart disease in literature (Table 2), there were two miRNAs significantly downregulated in plasma of Dachshunds with progression of endocardiosis. miR-30b was downregulated in ACVIM stage B dogs when compared to ACVIM stage A dogs (this miRNA also showed a downregulatory trend in ACVIM stage C dogs) and miR-133b was downregulated in ACVIM stage C dogs. These miRNAs could be potential diagnostic biomarkers of endocardiosis in Dachshunds. miR-30 and miR-133 are cardiomyocyte-enriched miRNAs which regulate connective tissue growth factor (CTGF)–a key molecule in the process of fibrosis and therefore an attractive therapeutic target of heart diseases. A decreased expression of these two miRNAs in human pathological left ventricular hypertrophy and rodent models of heart disease allow for the increase of CTGF levels, which contributes to collagen synthesis [33]. Downregulation of miR-30 is related to endoplasmic reticulum stress in cardiac muscle and vascular smooth muscle cells [34]. On the other hand the results of the study conducted by Goren et al. revealed elevated levels of miR-30 in the serum of stable chronic systolic heart failure patients [21]. The second miRNA with decreased levels in our study–miR-133 was also downregulated in human infarcted heart tissue [28]. Another study showed that the level of miR-133 in the plasma of patients with acute coronary syndrome was associated with high-sensitivity troponin T levels [35].

The next four analyzed miRNAs–miR-21, miR-29b, miR-125, miR-126 showed a trend of downregulation in the mild to moderate heart failure group–ACVIM B but the results did not reach statistical significance. The literature data for these miRNAs are present in Table 2. It is worthwhile to mention that during the study conducted on dogs by Steudeman et al. miR-21 was one of the 22 miRNAs that showed changed expression in the serum of Doberman Pinchers with Dilated Cardiomyopathy–a 1.67 fold change in microarray (without false discovery rate control), but the results of qPCR validation showed no difference between healthy and DCM dogs [29].

Although miR-208a and 208b are proposed as potential biomarkers of heart disease in humans [17,28,35] their expression has not been detected in any of the samples analyzed in our study. The reason might be that the etiopathogenesis of the most frequent heart disease in dogs-myxomatous valve disease may be different compared to humans [36]. Furthermore, an analysis of the heart transcriptomic profile showed a higher similarity between dog and mouse than between dog and human [37].

Moreover, levels of miR-423, which were elevated during left ventricular heart failure in humans [21,26], were equal in the healthy and diseased groups of dogs in our study. The results of the study conducted by Turatel et al. revealed that circulating miR-423_5p was not a useful biomarker for heart failure patients with a systemic right ventricle [38]. This means that we cannot consider miRNAs identified in one kind of heart disease as potential biomarkers of other heart diseases. miRNA patterns differed not only between species but showed different expressions of the same circulating miRNA within the same species and disease but different patient characteristics.

An important aspect of the study is a significant age difference between dogs belonging to different experimental groups. This is a natural phenomenon because the more advanced heart disease is always related to older age. The question is whether the age differences between experimental groups influence the findings of this study. This could be quite an important question since it is known that miRNAs may play a role in the age-related changes in the skeletal muscle phenotype [39]. In order to verify the presence of the age effect we decided to correlate the expression of miRNAs not only with the ACVIM stage of disease, but also with the age of the dogs. We created three groups consisting of the youngest, the oldest and middle age dogs. The results of this analysis (Additional file 1) did not show any significant changes in expression of miRNAs between these three different age groups. Thus we concluded that the cause of the changed expression of miR-30b and miR-133b in our study was the stage of the disease, not the age of the animals.

The utility of circulating miRNAs as biomarkers of many diseases has attracted considerable attention over recent years. However, it is also worthwhile to point out that the clinical application of miRNAs as biomarkers is still limited. One of the most significant obstacles is the difficulty concerning circulating miRNA normalization. Spiked in synthetic miRNA are widely used to normalize serum and plasma miRNA expression, but this approach does not include effects of pre-analytic variables on circulating miRNA measurement [40,41]. That is why we decided to use a previously described housekeeping gene in our study–miR-16-5p [42,43]. This miRNA is also a reference gene found on the Exiqon miRNome panels for plasma and is used as a control for hemolysis. Since the difficulties associated with hemolysis and platelet contamination of plasma samples are also significant, strategies were proposed to minimize the degree of red blood cell derived miRNA contamination, as well as minimize platelet contamination of plasma samples [44]. It must also be pointed out that the sole presence of specific miRNAs is considered to be a biomarker of disease, infection or inflammation. There are no measuring units, there are no possibilities of disease grading based on miRNA concentration and there are no reference levels of specific miRNAs in specific tissues, both healthy and diseased. Despite all these deficiencies, miRNAs are still very promising tools in the process of disease diagnostics.

## Conclusions

miR-30b could be a potential biomarker of ACVIM stage B heart failure in Dachshunds and miR-133b could be a potential biomarker of ACVIM stage C. The lack of expression of miR-208a and 208b in healthy dogs and dogs with heart disease and lack of significant changes in expression in the remaining 5 miRNAs which are potential biomarkers of heart diseases in humans proves that the findings in human medicine are not always directly reflected in veterinary medicine.

## Methods

### Study population

A prospective analysis and studies were carried out on 24 dogs submitted to the Cardiology Service of the Small Animal Clinic, Faculty of Veterinary Medicine, School of Agriculture in Warsaw. This study complies with national and institutional guidelines on the use of animals in clinical research according to the Polish legal act concerning experiments performed on animals of January 21st, 2005 (Ustawa o doświadczeniach na zwierzętach z dnia 21 stycznia 2005 r. (Dz. U. z 2005 r. Nr 33, poz. 289 z późn.zm.)). Since the blood for miRNAs analyses was taken during a routine veterinary examination and in accordance with the above mentioned legal act, a written ethical approval from the Local Ethical Committee before beginning the study was not necessary. However, before enrolling a dog into the study, an informed written consent for a full physical examination as well as additional diagnostic imaging tests and blood sampling via venepuncture was obtained from the owner and a high standard of care was adhered to throughout each examination. Study population characteristics are shown in Table 1 and blood test results in Table 3. Blood test results were not used as an exclusion criterion for this study. The majority of blood values were within reference ranges for the University laboratory or, where applicable, within published reference ranges. The values outside the reference ranges were minimal and the dogs were clinically healthy showing no signs of disease during the study, and so these values were deemed acceptable by the authors. Statistical analyses of clinical parameters were performed using GraphPad Prism version 5.00 (GraphPad Software, Inc., USA).

**Table 3:** **Blood test results of the study population in the control, ACVIM B and ACVIM C groups**

**ACVIM stage**	**ACVIM A–unaffected (mean ± SD)**	**ACVIM B (mean ± SD)**	**ACVIM C (mean ± SD)**	**Reference range**
PLT	272.5 ± 58.46	371.8 ± 95.81	472 ± 126.9**	200–600
Creatinine (mg/dl)	1.039 ± 0.14	0.9625 ± 0.13	0.92 ± 0.09	0.5–1.7
WBC (+10^9/l)	7.568 ± 2.21	6.498 ± 1.28	10.98 ± 3.92	5–17
RBC (+10^12/l)	7.4 ± 0.45	7.168 ± 0.47	7.559 ± 0.81	5.5–8.5
HGB (g/dl)	16.71 ± 1.13	16.11 ± 1.20	17.19 ± 1.95	12–19
HCT %	48.85 ± 3.12	48.02 ± 3.30	51.05 ± 5.91	37–57
MCV (fl)	66 ± 2.78	66.75 ± 3.20	67.43 ± 2.76	60–77
MCH (pg)	22.6 ± 0.89	22.46 ± 0.84	22.74 ± 0.63	19.5–24.5
MCHC (g/dl)	34.2 ± 0.72	33.54 ± 0.68	33.71 ± 0.91	30.8–35.9
RDW %	15.2 ± 0.58	15.36 ± 0.59	15.93 ± 0.61	13.4–18.1
Segmented neutrophils %	60.63 ± 5.63	70.63 ± 7.41	69.14 ± 7.08	51–85
Band neutrophil %	1 ± 1.60	2.88 ± 2.9	3.429 ± 3.64	0–3
Eosinophils %	7.375 ± 2.13	3.875 ± 3.27	6.857 ± 3.13	0–10
Lymphocytes %	28.5 ± 6.30	21.75 ± 7.40	17.86 ± 6.91*	8–35
Monocytes %	2.13 ± 1.25	0.63 ± 0.74	2.71 ± 1.89	0–10
Basophils %	0.375 ± 0.74	0.125 ± 0.35	0 ± 0	0–2
AsPAT (U/l)	30.93 ± 3.82	30.53 ± 5.72	35 ± 24.32	13–81
Urea (mg/dl)	28.13 ± 6.67	31.95 ± 6.46	27.97 ± 8.62	8–45
Total protein (g/l)	55 ± 3.51	55.13 ± 4.05	58.71 ± 2.69	50–83
Cholesterol (mg/dl)	207.1 ± 55.43	261.7 ± 39.9	267.7 ± 76.69	127.7–360
Ca (mg/dl)	9.638 ± 0.32	9.763 ± 0.56	9.829 ± 0.58	9.0–12.0
K (mmol/l)	4.639 ± 0.40	4.676 ± 0.39	4.854 ± 0.48	4.1–5.6
Na (mmol/l)	147.2 ± 2.43	150.2 ± 2.55*	148.9 ± 1.23	139.1–156.5
Cl (mmol/l)	111.3 ± 2.258	112.3 ± 2.872	106.5 ± 2.867**	98.7–118
HDL (mg/dl)	158.7 ± 30.05	183 ± 21.71	179.3 ± 28.75	205.3–111.0

*statistically significant difference vs healthy dogs with p < 0.05; **statistically significant difference vs healthy dogs with p < 0.01; ***statistically significant difference vs healthy dogs with p < 0.001.

### Clinical and laboratory examinations

24 client-owned dogs were examined cardiologically during routine veterinary examinations. The clinical picture of the animals included: animal history, clinical examination and echocardiographic examination. Dogs with recognized heart disease were classified according the ACVIM (American College of Veterinary Internal Medicine) classification scheme as stage B (asymptomatic)–8 dogs, stage C (mild to moderate heart failure)–8 dogs and stage A–8 dogs (the control group of unaffected dogs).

A complete physical examination was performed, with a specific emphasis on thoracic auscultation. A standard transthoracic echocardiographic (TTE) examination was performed in all dogs with the Aloka 4000 ultrasound machine, equipped with a cardiology program and 2.5–7-megahertz (mHz) sector transducers according to previously published norms. All examinations were performed at rest without pharmacological restraint. Blood samples for basic morphologic and biochemical analysis as well as the transcription profile analysis were collected from the jugular or cephalic vein. The following laboratory tests were performed in all of the dogs: complete blood count, serum sodium, potassium, calcium, urea, creatinine, albumin, liver enzymes, and cholesterol. The dogs were classified into groups based on clinical signs of heart disease and the results of clinical examination. Echocardiography exam was used to diagnose endocardiosis.

### Sample preparation and RNA isolation

Blood samples were collected in EDTA-K3 probes (Eqimed, Poland). Within 2 hours the samples were centrifuged at 1300 RCF for 10 minutes at room temperature. Afterwards the 250 μl plasma aquilots were transferred to 1.5 ml tubes (Eppendorf, Germany) and stored at–80°C until RNA isolation. Before extraction plasma samples were thawed on ice and centrifuged at 3000 × g for 5 min at a room temperature to pellet any debris and insoluble components. An aliquot of 200 μl of plasma per sample was transferred to a new microcentrifuge tube and the total RNA including small RNAs, were purified according to the protocol supplied with miRCURY™ RNA Isolation Kit–Biofluids (Exiqon, Denmark). In brief this procedures involves lyses of membranized particles using the provided Lysis Solution, precipitation of proteins and on column removing of any remaining impurities with provided Wash Solution. During isolation RNA Spike-in template mixture from miRCURY LNA™ Universal RT microRNA PCR, RNA Spike-in kit (Exiqon, Denmark) and carrier-RNA MS2 bacteriophage RNA (Roche Applied Science) were added as recommended by the manufacturer. RNA Spike-in kit was used for quality control of the RNA isolation and cDNA synthesis while MS2 RNA was used to minimize the loss of small RNA during isolation and the technical variation between replicates in further analyses. Total RNA was eluted by adding 50 μl of RNase-free water. The eluted RNA was stored at–80°C until further analysis.

### cDNA synthesis and real-time RT-PCR

cDNA was synthesized using the miRCURY LNA™ Universal RT cDNA Synthesis Kit II (Exiqon, Denmark). For quality control of this process UniSp6 Spike-in was used. The cDNA was stored up to 5 weeks in -20°C. Directly before preparation of Real-time PCR reaction the cDNA products were subsequently diluted 40×. All analyses were performed on individual samples using a SYBR® Green master mix, Universal RT (Exiqon, Denmark) as follows: polymerase activation at 95°C for 10 minutes; amplification (40 cycles) including denaturation at 95°C for 10 seconds, annealing at–60°C for 1 minute; melting curve including denaturation at 95°C for 0 seconds, annealing at 65°C for 15 seconds, continuous melting at 95°C for 0 seconds; cooling at 40°C for 30 seconds. All reactions were performed in triplicate and included the following controls: no template (RT NTC), no reverse transcriptase enzyme (no RT) and carrier MS2 RNA (carrier only). The primers are listed in Table 4. The amplification was performed in a Stratagene MX3005P in FrameStar 96 PCR plates (BioLab Innovative Research Technologies, Poland).

**Table 4:** **The sequences of miRNAs and accession numbers of the primers used in the study (Exiqon, Denmark)**

**Primer**	**Target sequence**	**Accession**
hsa-miR-126-5p LNA™ PCR primer set, UniRT	CAUUAUUACUUUUGGUACGCG	MIMAT0000444
hsa-miR-208a LNA™ PCR primer set, UniRT	AUAAGACGAGCAAAAAGCUUGU	MIMAT0000241
hsa-miR-208b LNA™ PCR primer set, UniRT	AUAAGACGAACAAAAGGUUUGU	MIMAT0004960
hsa-miR-423-5p LNA™ PCR primer set, UniRT	UGAGGGGCAGAGAGCGAGACUUU	MIMAT0004748
hsa-miR-133b LNA™ PCR primer set, UniRT	UUUGGUCCCCUUCAACCAGCUA	MIMAT0000770
hsa-miR-125b-5p LNA™ PCR primer set, UniRT	UCCCUGAGACCCUAACUUGUGA	MIMAT0000423
hsa-miR-30b-5p LNA™ PCR primer set, UniRT	UGUAAACAUCCUACACUCAGCU	MIMAT0000420
hsa-miR-21-5p LNA™ PCR primer set, UniRT	UUCCCUUUGUCAUCCUUUGCCU	MIMAT0000668
hsa-miR-16-5p LNA™ PCR primer set, UniRT	UAGCAGCACGUAAAUAUUGGCG	MIMAT0000069
hsa-miR-29b-3p LNA™ PCR primer set, UniRT	UAGCACCAUUUGAAAUCAGUGUU	MIMAT0000100

### Data analysis

Initial qPCR data analysis was performed using MXPro software (Stratagene). Threshold cycle (Ct) for each miRNA was extracted by setting a threshold and automatically defined baseline. Furthermore, the data were analysed statistically using Exiqon GenEx qPCR analysis software (Exiqon, Denmark). miR-16-5p was used as a housekeeping gene [42,43]. This miRNA is also a reference gene found on Exiqon miRNome panels for plasma and is used as a control for hemolysis. The fold change for each miRNA was calculated using ΔΔCt method [45]. Data are shown as means ± SEM.

## Additional file

Additional file 1:
**The expression of miR-21, miR-29, miR-30b, miR-133b, miR-126, miR-423 and miR-125 in dogs with heart failure divided into groups based on age (fold changes relative to youngest group; mean ± SEM).** Dogs were classified into three groups as follow: group 1: 42.29 ± 21.91 months (mean ± SD, n = 7); group 2: 102.1 ± 14.57 months (mean ± SD, n = 8); gr 3: 157.6 ± 7.927 (mean ± SD, n = 8).
