# Supplementary material for: Plasma miRNAs as potential biomarkers of chronic degenerative valvular disease in Dachshunds
Source: BMC Vet Res. 2014 Sep 26;10:205. doi: 10.1186/s12917-014-0205-8 (PMC4193998; doi:10.1186/s12917-014-0205-8)
Supplement: Additional file 1: — The expression of miR-21, miR-29, miR-30b, miR-133b, miR-126, miR-423 and miR-125 in dogs with heart failure divided into groups based on age (fold changes relative to youngest group; mean ± SEM). Dogs were classified into three groups as follow: group 1: 42.29 ± 21.91 months (mean ± SD, n = 7); group 2: 102.1 ± 14.57 months (mean ± SD, n = 8); gr 3: 157.6 ± 7.927 (mean ± SD, n = 8). [file 12917_2014_205_MOESM1_ESM.docx]

**Age: group 1 vs 2**

**Age: group 1 vs 3**
